# Regional Regulations and Public Safety Perceptions of Quality-of-Life Issues: Empirical Study on Food Safety in China

**DOI:** 10.3390/healthcare8030275

**Published:** 2020-08-15

**Authors:** Guanghua Han, Simin Yan, Bo Fan

**Affiliations:** School of International and Public Affairs, Shanghai Jiao Tong University, Shanghai 200030, China; ann1312911@163.com (S.Y.); fanbo@sjtu.edu.cn (B.F.)

**Keywords:** food safety, perceptions, local governance, administration, food safety inspection

## Abstract

In many developing countries, the public is shifting its focus from economic growth to quality-of-life issues. As a result, there is extensive demand for better public administration of quality-of-life issues, ranging from air pollution to food safety problems, that threaten ordinary peoples’ health and daily lives. This article analyzes the determinants of public perceptions of food safety and the administrative effects of regional governance in different provinces with nationwide survey data. A two-level hierarchical linear regression model (HLM) with provincial factors as background-level variables and demographic factors as individual-level variables was developed to measure the influence of these factors on public perceptions of food safety. The results showed that female, young, and well-educated urban residents perceived greater risks to food safety than other groups. Administrative fiscal expenditures and local normative documents in different provinces did not have significant effects on public perceptions of food safety. However, food safety inspections weakened public perceptions of food safety. We thereby suggest that provincial governments invest in more efficient food safety projects and enhance the publicity of normative documents in popular media.

## 1. Introduction

With economic development, the populations of developing countries are becoming less satisfied with the basic provision of services and shelter by governments; people also expect better public administration regarding quality-of-life issues, including food safety. The perception of food safety is a subjective psychological state that reflects people’s confidence in food safety. As the largest developing country in the world, China has a population of more than 1.4 billion and it is extremely important to mitigate all risks, including food safety risks, in society. China has had many food safety scandals over the past decade, particularly the cases of toxic Sanlu milk powder in 2008 and past-date Fuxi meat in 2014, and these substantially damaged the credibility of the entire food industry and negatively influenced public perceptions of food safety [1,2]. Food scandals have evoked widespread social concern and extensive panic across various modern social media platforms for many years. For example, the scandal involving melamine-tainted milk powder produced by Sanlu was a serious food safety incident in 2008, which evolved into a national dairy-industry crisis in China. Almost 10 years have passed and people’s anxiety about these risks remains. Because they are wary of the safety of milk powder, an increasing number of Chinese parents are choosing foreign-brand or Chinese-foreign joint-brand infant milk powders and the proportion of families making this choice has increased in main cities by up to 80% in recent years [3]. Therefore, identifying perceptions of food safety and the determinants underlying those perceptions is essential to the strategic formation of governmental policies and the mitigation of public anxieties about food safety.

There are significant cultural differences throughout the world in the production, serving, and consumption of food. The traditional Chinese saying, ‘Men work outside the home, while women work in the home’ expresses a unique aspect of Chinese culture. In traditional Chinese families, especially in rural areas, adult men are generally responsible for external affairs, while women perform household duties. Compared with Western countries, Chinese people have different eating habits. For example, Chinese people prefer to consume fully cooked foods and engage in group-dining with shared plates. Accordingly, existing studies have observed that the determinants of food safety perceptions are not identical across different countries. For example, women have been found to express higher levels of concern and anxiety than men about food safety risks in America [4], but an empirical study in the Chinese context indicates that gender does not make any difference in public perceptions of food safety [5]. Similarly, families with underage children tend to perceive a higher risk to food safety in the Canadian province of Alberta [6], while that relationship was not found to be significant in a survey conducted in 30 major cities in mainland China. The conflicting findings in these studies are enlightening and reveal that the variation in individual perceptions food safety status quo, and subsequent behaviors may also be influenced by regional culture and social customs. We wondered whether perceptions of food safety were the same across different areas of the same country when those areas had different economic and managerial configurations. A national survey by the ABS (the Asian Barometer Project Office (ABP Office) is solely responsible for data sharing and usage; we appreciate the support of the ABP Office in providing the data and the authors take full responsibility for the views expressed in this study) that generated 3346 complete and valid responses suggests that the average public perception of food safety status quo in mainland China is 5.509. The scores range from 0 to 10; the most positive attitude is 10. We also found an internal difference in food safety perceptions between provinces (Table 1). Two aspects of the survey demand attention. On the one hand, the residents of 23 of 26 provinces perceived food safety as poor (below 6 scores), revealing the general public’s widespread anxiety and dissatisfaction regarding food safety. On the other hand, there exists considerable regional diversity with regard to perceptions of food safety. For example, the most positive score was 6.888 from Yunnan province and the most negative score was in Hebei province, with a score of only 4.196 points.

According to classic theories such as resource-dependence theory, public perceptions of food safety status quo are possibly directly affected by government resource inputs into regulation. Specifically, more regulation input theoretically generates better management performance, which in turn enhances public perceptions of food safety. Since the conventional proposition has rarely been empirically tested, it is theoretically imperative, as well as practically important, to examine regulatory resources for public perceptions of food safety. Regulation resource input is characterized by indicators such as political efforts, enforcement actions, and fiscal expenditures on food safety regulations. Since the factors differ considerably in terms of integrity and validity in different areas, investigating the influences of regulatory resources also enriches managerial insights and has political applications. Resources are the key ingredient of regulatory legitimacy and food scandals and media amplification of food safety risks have encouraged government authorities to invest more on regulation. However, resources may not automatically transform into governance capacity, which is directly linked to residents’ perception in daily lives. Since existing studies seldom examine the connection between governance resource and its results of people’s perceptions, we examined the effects of regulatory resources on public perceptions of food safety. As one of the most concerning health-related issues in people’s daily lives, food safety attracts the ubiquitous attention of the general public. Our hypotheses were tested quantitatively with a nationwide survey across 26 provinces in China. The results suggested that regulatory resources are pivotal in shaping public perceptions of food safety, which sheds light on the theory of social risk management. We suggest that provincial government agencies strengthen food safety-related information disclosure and enhance readability for the general public in a regulated way. We also recommend that provincial governments pay special attention to fiscal expenditure deployment, which reduces physical food safety risks, decreases food scandals, and lowers public negative perceptions regarding food safety.

## 2. Theoretical Framework and Hypothesis

### 2.1. Food Safety Perceptions and Determinants

A review report regarding people’s purchase decisions by Smith and Riethmuller [7] suggested that consumers’ behavior is first determined by subjective perceptions of risk rather than other issues (e.g., quality, price, package [8]). Slovic et al. [9] designed indicators to measure respondents’ perceptions of food safety, where risk controllability, severity of consequences, the attribute of risk delay, and knowledge of risks were included as indicators. Some existing studies have attributed people’s perceptions to their emotions under certain risk exposures. It has been found that the most significant emotion that might affect people’s perceptions is ‘anger’ [10].

With the development of the economy, the general public is not merely concerned with basic needs such as foods and shelter but also has considerable requirements for better quality-of-life issues, ranging from air/land pollution to water/food safety [11,12]. Since quality-of-life issues threaten peoples’ health and daily lives, the general public expects effective governmental administration to mitigate risks, and the public has expected the government to exert more pressure on the administration of environmental protection in recent decades [13]. As a developing country, China is a typical case for exploring residents’ emerging concerns about quality-of-life issues and public administration. In the past few decades, China has achieved rapid economic growth and has been confronted with a series of challenges such as environmental destruction and food safety [14]. In fact, food safety has become a critical threat to peoples’ health. Several food safety scandals have been reported, including contaminated baby formula, rice with excessive cadmium, and cooking oil recycled from waste oil. As a result, Chinese residents have become aware of food safety [15,16,17]. A survey by Pew Global Survey showed that 71% of Chinese residents believe that food safety is one the most urgent problems in need of solving [18,19]. Food safety issues have resulted in national anxiety about food safety risks and eagerness for the continuous improvement of public administration [5,13]. These facts motivate us to explore whether provincial regulations mitigate food safety anxiety among the Chinese public. Previous studies investigated perceptions of food safety from two perspectives: the perspective of individuality and the environmental perspective.

Some scholars have found that perceptions of food safety risk are closely linked with socioeconomic status and demographic characteristics [20]. Thus, some studies have focused on the relationship between demographic factors and perceptions of food safety [21] and the influences of socioeconomic factors on respondents’ perceptions of food safety [22,23,24,25,26]. Some studies observed the influences of demographic factors (e.g., gender, age, educational level, income, marriage) and socioeconomic factors (e.g., consumers’ knowledge of policy, media exposure, information pattern, state employment) based on data from different countries or regions. Since the same factors may function differently with regard to public perceptions of food safety in different social or cultural circumstances, we considered demographic factors as control variables and examined their influences on perceptions of food safety in the Chinese context.

Another stream of literature has emphasized the influence of social environment and culture. Yeung et al. [8] attributed public perceptions of food safety to three sources, i.e., bacteria, chemical additives and new technologies such as transgenic technology. Moreover, media reports [27] a risk prewarning system [28] and information communication [29] significantly affect public perceptions of food safety. A study conducted by Hohl et al. [30] with data from 25 European countries identified the three most important exogenous factors and revealed that cross-national diversity may contribute to different perceptions of food safety. Provincial characteristics (e.g., economic development, government capacity for administration and publicity regarding policies) vary throughout China, which may lead to provincial differences in public perceptions of food safety.

Meanwhile, previous studies have indicated that public trust in government is a crucial determinant of residents’ perception of food safety [30,31]. Moreover, trust in government authorities and food producers have significant impact on public confidence in food safety status quo [32,33,34]. On the other hand, lack of political trust is the most possible reason for failure risk communication in aligning official and public standpoints [35]. Some food safety incidents in China, Shanlu powder scandal for example, indicates that food safety scandals exacerbate public distrust in government and sap public confidence in the government’s regulatory capacity in return. Evidence suggests that the Chinese public, as well as many scholars, have attribute food safety scandals to poor regulation of food markets and appeal government for regulatory reforms [36,37].

Although regulatory compliance through regulatory authorities has been well addressed in a few studies, the link between it and public perception about food safety has not been systematically examined. A recent study by Ma and Liu [38] empirically examined the effect of regulatory resources including manpower, fiscal revenue and food scandals on people’s perception about food safety risk and explored the missing linkages among them. They found that sufficient fiscal revenue and manpower do not elicit more favorable public perceptions of food safety, which could be attributed to ineffective distribution and deployment of resources. On that basis, we examined whether regulatory measures such as provincial normative documents, sample inspections, and fiscal expenditures on food safety, work to mitigate public food safety risk perception. Specifically, this study aimed to empirically examine the effects of provincial regulatory measures on perceptions of food safety and propose explanations.

### 2.2. Provincial Regulations and Public Perceptions of Food Safety

A well-known study by Slovic et al. [39] categorized general factors that determine public risk perception into three categories: controllability of risks, familiarity with risks, and risk exposure. Thus, regional differences in perceptions of food safety may be explained by different managerial resources and regulatory activities in different areas [40].

Due to information asymmetry regarding food quality between food producers and society, risk communication has a significant capacity to shape perceptions of risk [41]. Existing research indicates that risk communication by equitable information is regarded as important, so that the public may prepare for and deal with risky situations that arise in daily life. In the risk communication process, institutional trust contribute to develop more efficient risk communication. Since public incompetence of information processing, trustworthy government documents provide creditable information regarding social risk and regulation statue [42]. Information publicity about risk, what is reported, to whom and by whom, and by what method is crucial to risk perception. Therefore, the constructive nature of risk provides opportunities for government to mitigate public risk perception of food safety. According to the Government Information Disclosure Bill and Food Safety Law of the People’s Republic of China, normative documents that report the results of food safety inspections should be disclosed to the general public [43]. Because of the information asymmetry in food production, normative documents enable the public to know more about food processing and food quality control systems [44]. Additionally, normative documents make a clear recommendation for risk protection which public concerns [45]. Therefore, normative documents are assigned as a convenience to accomplish a communication that required by regulation [46]. Apparently, governmental regulation efforts could be specified in normative documents [47], and regulatory policies can enhance public perceptions of risk controllability with regard to food safety. Hereby, we present hypothesis H1.

The public recognizes the risks of subjective understanding, which is common sense with regard to people’s own prevailing protection. Thereby, there exists gaps in risk perceptions between the public and government [48]. Risk controllability indicates people’s psychological predilection to protect themselves and avoid danger [8], and is the most critical factor that determines public perceptions of risk. In other words, people’s perceptions become more negative with their sense of risk controllability, and an enhancement of the sense of risk controllability can effectively reduce people’s fears. In practice, both direct (e.g., checking food labels, purchasing foods from good reputation producers) and indirect (e.g., control guarantees by government monitoring and regulatory system) drive enforces to food safety perception [49]. Residents’ familiarity with risks regarding food safety, which includes risk identification and understanding food hazards, is related to government regulatory activities. Being familiar with food safety risks is helpful for accurate risk identification. From a cognitive perspective, people who have more risk knowledge could more accurately identify risks, resulting in their having fewer fears regarding food safety risks [50]. Thus, food knowledge and risk information shared by government regulators can improve public familiarity with food safety risks and potentially mitigate public perceptions of food safety. The China Food and Drug Administration (CFDA) promulgates a food information regulation called “Administrative Measures for Sampling Tests of Food Safety”. The regulation requests that provincial food administration offices establish food sampling tests and an administration system and regularly supervise the safety of food in the market. Most importantly, the mechanism requires safety testing information and standards to be publicized. Through this information mechanism for publicizing sampling test results, the public gains access to information about risk resources, hazards, and probability of occurrence, as well as food safety standards and consumption guidance. Risk communication provides transparency in regulation and increases the public’s trust in governments, as well as helps reduce people’s risk perceptions [51]. As a result, sample inspections and the corresponding information sharing contribute to public access to information and knowledge about food safety risks. Meanwhile, sharing sample inspection information helps the public to know more about food safety risks and enhances the public’s familiarity with food safety risks. According to theories of perception [52], people’s anxiety decreases as their familiarity with risks increases and publicizing the results of sample inspections can mitigate negative public perceptions of food safety (hypothesis H2).

Risk cannot be perceived unless people are exposed to risk and believe it exists [27]. Modern media and social networks increase the possibility of exposure to food safety scandals. Many food issues are widely known within hours of the news being posted on the internet. Sheltering residents from threats and panic is one of the government’s responsibilities. Apart from personal psychological factors, governance capacity is a pillar of crisis management [38]. Efficient regulation of food safety require sufficient resources, including financial investment, technologic equipment, and personal involvements. As a result, it is theoretically important to examine the impact of government regulatory resources on residents’ perceptions of risk. According to resource-dependence theory, organizations and companies must access to external resources to perform its functions, and without the resource, they are not able to complete work activities [53]. The success of organizations and companies relates to the scarcity of the input resources, which thereby has a fundamental impact on success. Regulatory resource are key inputs to foster professional manpower resources and regulatory capacity. Therefore, regulatory resources by fiscal expenditure of food safety can help shape peoples risk perception in several ways. Government authorities usually have the resources to address risks and mitigate residents’ perceived risks through many governmental activities ranging from communal participation and prewarning systems to knowledge trainings [54]. Additionally, abundant fiscal expenditures help imply food safety projects, such as food safety monitoring equipment, food safety accidents early warning system, and preparation of food safety crisis. Existing studies suggest that sufficient budgetary resources are important for strong governance capacity and regulatory effectiveness [32], which significantly increases public satisfaction and reduces public anxiety of food safety. Thus, we employ provincial per capita fiscal expenditure on food safety to indicate provincial inputs on food safety regulations and propose the following hypothesis H3.

**H1.**  *Higher volumes of provincial normative documents about the supervision of food safety enhance positive public perceptions of food safety*.

**H2.**  *The number of food inspections by provincial governments is negatively associated with levels of public perception of food safety*.

**H3.**  *Higher provincial expenditure on food safety contribute to positive perceptions of food safety*.

Based on state regulations, provincial governments make efforts to regulate food safety at the regional level. In this paper, we examined the effects of provincial regulations through normative documents, food sampling inspections, and fiscal expenditures on public perceptions of food safety.

As a subjective psychological phenomenon, safety perception is closely related to personal demographic factors. To analyze the determinants of people’s perceptions of food safety, it is necessary to include demographic characteristics in the analysis model as explanatory variables. As mentioned above, there are a few existing studies that have indicated that demographic characteristics (e.g., age, gender, marriage and child status, education level) may exert significant impacts on residents’ perceptions of food safety [55]. In contrast to males, females come in contact with food more frequently, are more concerned about food safety, are more afraid of food safety risks, and usually have higher risk perceptions of food safety [6]. In comparison with young consumers, older consumers have abundant life experience, are generally equipped with more knowledge about food safety, have greater familiarity with food risks, and usually have worse perceptions of food safety [56,57]. Married people and families with children have to be concerned about the health of their family members and are more sensitive to information about food safety incidents. They fear food risks and thus may have more negative perceptions than those who are unmarried or have no children. Compared to less-educated consumers, well-educated consumers pay more attention to media coverage of food safety incidents and healthy lifestyles, and they usually have more negative perceptions [58]. In addition, as the food processing and service industries are more developed in cities and towns, urban residents are more likely to face food safety incidents than those in rural areas. Meanwhile, the risks are more widely dispersed. Therefore, urban residents may have more negative perceptions of food risks. Based on this analysis, research hypotheses regarding the impacts of demographic-level variables on perceptions of food risks are put forward. We controlled the demographic and socioeconomic variables that may affect public risk perceptions in our models.

Since the factors included in the study were at the individual and provincial levels, a hierarchical linear regression model was adopted to identify the determinants of perceptions of food safety at different levels. In this study, we mainly focused on exploring the provincial effects on respondents’ perceptions of food safety. Since there was very little evidence or theoretical expectations indicating that provincial regulation moderates the relationship between demographic factors and perceptions of food safety, cross-level effects were not examined in this study.

## 3. Data and Methods

The data employed in this study were collected by the Asia Barometer Project in its fourth round of the Asian Barometer Survey (4th ABSs). The survey, which involves a questionnaire consisting of over 200 questions, was conducted by face-to-face interviews from July 2015 to March 2016. In the survey, a total of 125 primary sampling units (PSUs) were used based on the Census Yearbook from the National Bureau of Statistics of China and respondents were randomly selected from the PSUs with a representative sampling method of probability proportional to size (The sampling method uses the “GPS Assisted Area Sampling Method”, which incorporates population as a measure of size, stratification, and multi-stage PPS (probabilities proportional to size). The sampling method was created by the Research Center for Contemporary China (RCCC) at Peking University; a full description is available at Landry, F. Pierre and Shen Mingming [59]. The target population included Chinese people aged 18 and above who had the ability to respond and had resided in the surveyed area for at least one month. Additionally, people who resided in certain places were not considered in the survey, i.e., military residential complexes, residential units in compounds of central ministries, embassies and consulates, infrastructure buildings such as power stations and water stations, prisons, tourist destinations and religious sites. A total of 6013 eligible samples were drawn in the field. After the interviews were completed, three rounds of validity checks were undertaken on every questionnaire by the interviewer, a supervisor and a data manager in the ABS central office. The standard for the validity check included the use of the correct process for reaching the target interviewee and the use of the standard interviewing process, the ability of the interviewee to understand and answer the questionnaire, and the reliability of the interviewee’s response. In this study, we included six demographic variables in our analytical model. In the end, we further excluded from the questionnaire incomplete questions regarding our demographic variables. Ultimately, 3346 complete and valid interviews were obtained in mainland China. In this study, we employ the completed and valid data at level 1 in our hierarchical regression model.

The provincial normative documents included effective food safety-related regulations and laws issued by each provincial government and the provincial PC (People’s Congress) and FDA (Food and Drug Administration) agencies. The subjects of the normative documents, which included risk monitoring, accident settlement, standards for production, operation, supervision, and management, were counted in the dataset. Since the 4th ABSs in mainland China were finished in March 2016, we collected the regulations and laws before that date. The normative documents provide the basic standard guidelines for food processing and safety, and these measure provincial governments’ regulatory policies on food safety. In addition, the food and drug administration (FDA) office in each province inspects food safety in the market by sample inspection and publicizes the sampling test information. The frequency of inspection indicates the province’s efforts at food market supervision. We collected inspection information from supervised sample inspections and evaluations of sample inspection made by province governments and provincial FDA authorities in 2015. The sample inspection information was available on the websites of provincial FDA authorities. The provincial fiscal expenditures of each province in 2015 were drawn from the Bureau of Statistics for each province.

The demographic explanatory variables include individual characteristics such as gender, age, marriage, child status, education, and place of household registration, which come directly from the research questionnaires. The demographic characteristics of the sample are statistically described in Table 1. In terms of the frequency and percentage data, the male and female respondents were evenly distributed, each accounting for about half. In terms of age distribution, a certain number of respondents were covered in all age groups, with relatively older respondents as the largest percentage. In addition, the majority of respondents were married without children, were less-educated, and had a rural household registration. The numbers of people in each category are summarized in Table 2.

The background explanatory variables represented relevant indicators for the food safety affairs of units at the provincial level (as shown in Table 2), including the number of provincial normative documents, the number of annual food safety inspections, and fiscal expenditures (per capita). Data for the background variables covered a total of 26 provincial administrative units, excluding Tianjin, Qinghai, Ningxia, Tibet, Xinjiang, Hongkong, Macao, and Taiwan. According to the statistical results, the quantity of regulatory documents on food safety formulated by the provincial regulators differs greatly. The region with the lowest quantity had only one provincial normative document and the region with the most quantity had issued 32 normative documents. The difference in regulation intensity was also represented in the number of sample inspections conducted by the provincial FDA annually: a minimum of 1152 inspections, a maximum of 32,594 inspections, and an average of 8885 inspections for all provinces. In addition, provincial fiscal expenditures (per capita) range from 137 RMB to 7309 RMB, with an average of 1494.38 RMB. Based on these descriptive statistics, it can be seen that the regulatory policies on food safety and fiscal investments also differ greatly among provinces. Since the food-safety regulations of authorities can be divided into routine supervision and incident punishment, normative documents and regular sample inspections are the major patterns and routine approaches that provincial authorities use to supervise local food markets, and these variables intuitively reflect the effectiveness and validity of the official supervision of food safety. These data were available through the official website, so the statistics are solid and reliable, in contrast with data on official punitive actions related to illegal food enterprises, as those data are opaque and unstructured. Thus, it is relevant to consider the data on provincial normative documents and sample inspections as background-level independent variables (Table 3).

The dependent variable refers to residents’ evaluation of the status quo of food safety, which was mainly measured by the question: “What do you think about the current status quo of the following aspects in China?” in the questionnaire survey. This question was scored from 1 to 10 to evaluate nine specific issues, including the evaluation of current food safety, with 1 point indicating very poor status quo of food safety, and 10 points indicating very good status quo. The higher the score, the more optimistic the public felt about food safety in China and the lower their perceptions of risks. The general average score for such evaluations was not high, only 5.41, reflecting to a certain extent residents’ low level of satisfaction with the current status quo of food safety in China. The highest evaluation score was 6.65, on average, and the lowest was only 4.32, which indicates a great difference in citizens’ perceptions of food safety in different regions.

## 4. Model and Analysis of Results

Since each respondent belongs to a certain province and because every province has many respondents, the data used in this study have a typical nested structure where individuals nest in provinces. Because the individuals in each province are under the same provincial regulation system, they are not independent from each other, which violates the theoretical assumptions of ordinary least square models. Therefore, hierarchical linear models (HLMs) were more appropriate for this analysis [30]. We employed an HLM to estimate the influencing effects of provincial-level regulatory factors (Level 2) by controlling for demographic and socioeconomic variables at Level 1. The model analysis was divided into three steps. First, a null model was established to verify the explanatory power of the background-level variables to judge the applicability of the hierarchical linear regression model (Model 1). Second, a random-coefficients regression model was established to determine the direct effect of demographic variables on perceptions of food safety and eliminate insignificant indicators (Model 2–3). Finally, a complete model was established to analyze the direct effect of the background variables (Model 4).

Model 1 in Table 4 shows the analysis results from the null model. The results indicated that the estimated value of the individual-level variance (valued 6.11 in Table 4) was much larger than that of the background-level variance (valued 0.21 in Table 4). Thus, the model’s interclass correlation (ICC) equaled 0.034, which suggested that 3.4% of the difference in residents’ perceptions of food safety results from differences in provincial-level factors. Although the proportion of variance explained by provincial-level variables was relatively small, the likelihood ratio test (χ^2^ = 126.11, *p* = 0.00) suggested that the background provincial-level factors significantly influenced people’s perceptions of food safety. We then introduced all control variables in an HLM (Model 2). The results suggested that highly educated, urban-registered, young, female residents tend to perceive food safety more negatively than others, while the number of children in the family did not significantly affect respondents’ perceptions of food safety.

Most of the empirical results in Model 2 were consistent with existing studies [60] and explainable. First, the gender variable coefficient was positive. This indicated that compared to females, males had a positive evaluation of the status quo of food safety and negative perceptions. Females feared food safety risks more and had more negative assessments of the food safety status quo mainly because they are often meal planners in families [61] and more vigilant about food-related risks caused by bacterial contamination, pesticide residues, and additives. Second, the value of the age coefficient was positive. This indicated that when compared with older respondents, younger consumers usually had worse evaluations of the status quo of food risks and had negative perceptions of food risks mainly because the younger consumers usually had less knowledge of food safety, less understanding of the information about food risks, and felt more vulnerable to negative media reports and social panics. Therefore, they often had positive perceptions of the food safety status quo. Third, the coefficient of education level was negative. This reflects that respondents with more education had more negative evaluations of the status quo of food safety risks than respondents with less education. The higher educated groups were more concerned with their own dietary safety, more sensitive to media reports on food safety incidents. and often had more positive perceptions of food safety. Fourth, the value of the coefficient of marital status was negative but not significant, which consisted of a survey by Lee et al. [62]. This indicated that when compared with the unmarried and single people, married and cohabiting respondents did not perceive more food safety risks. Some prior studies found that marital status did not affect food safety behaviors [63], which explains the insignificant influence from marital status. Fifth, the coefficient of urban and rural residents’ perception was negative. This indicated that urban residents have more positive perceptions of food safety and more negative evaluations of the status quo of food safety than rural residents. Although the food processing and service industry was far more developed in cities than in rural areas, the probability and frequency of food safety incidents were much higher in urban areas than in rural areas. Thus, urban residents were more exposed to food risks and therefore had more positive perceptions on food safety. In addition, the statistical results for number of children was not significant, which may be attributed to the age distribution of the respondents. As more than 45% respondents were more than 50 years old, their children were mostly adults and had formed new families. Thus, the respondents did not worry too much about the food safety of their adult children. Therefore, the number of children had no significant effect on the respondents’ perceptions of food safety.

We then included the variables at level 2 together with those at level 1 and obtained a complete model (model 3–5). First, the number of food safety inspections conducted by the provincial FDA had a significant effect on residents’ perceptions of food safety (*p* value < 0.05). The positive coefficient (0.222) indicated that the more annual sample inspections of food conducted, the higher scores residents gave food safety and the more positive perceptions of food safety they had. This result verified research hypothesis H1, reflecting that more sample inspections of food were held and the publicizing of this information in a more frequent manner made it easier for residents to obtain knowledge and information about food safety. This indicated that food safety inspections and supervision measures adopted by provincial food regulators have increased public familiarity with risks and are conducive to improving perceptions of food safety. Second, the relationship coefficient between the number of provincial normative documents and the current evaluation of food safety (−0.016) was negative, and the relationship coefficient between the provincial fiscal expenditure (per capita) indicator and the evaluation coefficient (0.126) was positive, but the regression coefficients were both not significant. Contrary to our expectations, provincial fiscal expenditure per capita and normative documents did not significantly affect respondents’ perceptions of food safety. Thereby, assumptions H2 and H3 were not supported by the empirical results.

## 5. Discussion and Conclusions

We first discuss the key findings from our empirical analysis and then discuss the policy implications. Finally, the study limitations and suggestions for future research avenues are presented to conclude the study.

### 5.1. Discussion

Regional governments implement national policies and take responsibility for governance in the regulatory arena. Through centralized national policies, regional governments take charge of local issues with additional regulatory resources, such as regional input, law enforcement, and administrative regulations. The provincial resources of regional governments can help mitigate public negative perceptions of safety and prevent social panic, but such claims have not been empirically tested in the past. Therefore, we employed three measures of provincial resources including normative documents, food safety inspections, and fiscal expenditures (per capita) to explore the effectiveness of provincial resources on residents’ perceptions of food safety, which has implications for improvements to regulatory policy and government administration. We found that the resources of normative documents and fiscal expenditures did not significantly shape public safety perception. Consistent with our expectations, sample inspections of foods had significant effects on public perceptions of food safety. Specifically, provincial sample inspections were related to positive perceptions of food safety. Compared with provincial factors, individual demographics and socioeconomic characteristics were underpinning factors that affected public perceptions of food safety.

Resources are the key ingredient of regulatory legitimacy [64]. Food scandals and media amplification of food safety risks have encouraged government authorities to invest more fiscal expenditures on regulation. However, fiscal expenditures invested in food safety regulation may not automatically translate into government regulatory capacity, which is the crucial component of the social psychology of safety perception [65]. Resources are invested without contributing to governmental capacity building, and effective allocation matters in determining the effectiveness of the input in many complicated contexts [38]. In other words, public perceptions of food safety cannot be mitigated as long as the resources invested do not contribute to governance capacity. Therefore, it not surprising that hypothesis H1 was not supported in our empirical study.

In fact, the missing link between fiscal expenditures and governance capacity can be explained by many underling reasons. If we track the flow of fiscal expenditures, we find the fiscal expenditures are mostly distributed to equipment purchasing and maintenance, reagents and consumables. The outputs of fiscal expenditures are used to regulate business operations in food production and processing under legal specifications, where administrative sanctions and stipulated penalties are familiar to the general public. On the other hand, many foods are produced and regulated in one province but sold in another. As a result, some proportion of resources does not translate into regulatory capacity for the local food-consuming market in provinces. To mitigate residents’ anxiety about perceptions of food safety, resources on local food regulation could be more comprehensive and publicized more effectively. In fact, widespread food safety scandals have posed serious distrust of food producers, retailers, and regulatory authorities. As a result, many Chinese public have attributed food safety scandals to lax enforcement and poor governance, which often leads to public suspects of official corruptions and misconduct in building governance capacity with huge of fiscal expenditures [66,67]. Therefore, reducing information asymmetry via transparent information transmission, media monitoring, and public participation is helpful to rebuild political trust and then enhance their confidence on investment efficiency of food safety regulation [68]. Since efficient investment on regulation enhance controllability of food risks, the public likely has less negative perception of food safety in return.

Sampling inspection plans aim to assess the “fitness for use” of batches of foods, which is particularly popular in many parts of society. Food sampling inspections are widely used in China’s FDA, and the empirical conclusions suggest that sampling tests significantly reduce residents’ negative perceptions of food safety. Risk perception theory claims that risk controllability and risk familiarity are key factors in people’s psychological state of risk perception. Risk communication throughout the risk analysis process, control measures, and information feedback systems bridges the gaps between regulators and the general public. Sharing food safety inspection information helps to warn the public and guide them in how to deal with risks [10]; risk communication has become an essential measure of risk perception. In practice, administrative measures for food safety sampling inspections have been implemented in China and authorities are required to make sampling results public in a timely manner through mass media (e.g., government websites, online media, TV and broadcasts, newspapers). This is an effective part of the current efforts to mitigate residents’ negative perceptions of food safety. Considering the growing interest in civic engagement in the regulatory process in recent years [69], government response can be taken as a regulatory capacity. Thus, designing and establishing provincial response mechanisms could be an effective institutional innovation for promoting positive perceptions of food safety. Theoretical propositions have been used to examine and support qualitative analyses, thereby enriching the literature. Two-way communication can be extended both in practice and through theoretical studies in the future.

Contrary to our expectations, the provincial normative documents enacted by provincial government authorities were not significant to public perceptions of food safety. An important and possible explanation can be attributed to the accessibility and readability of normative documents. In the past few years, provincial governments have made efforts at data sharing (through e-government sites) with internal government authorities and have attempted to provide citizen-centered public service through information and communication technology. Most provincial normative documents are publicized on provincial websites to residents. It was reported that the number of government websites was 84,094 in 2015 when the survey data we used were collected, which included more than 2998 provincial websites [70]. Consistent with some prior studies, a proportion of provincial governments could not be accessed in third-party testing [71].

At the end of 2015, approximately half of Chinese residents had access to the internet through computers and cellphones [72]. However, less than 25% of internet users had used online governmental services, among which only 20% had accessed government websites. We used the Beiijng governmental website as an example. There were only 13,800 visitors to the Beijing governmental website every day on average in 2015, while there were 21,705 million people residing in Beijing at that time. In other words, every resident visits the Beijing website once every 4.3 years. Since Beijing is one the most developed areas in mainland China with the highest rate of internet penetration, the click-through rate of other provincial websites should be much lower. This indicates that few residents have accessed provincial normative documents and further explains why the provincial normative documents publicized on provincial websites typically do not significantly affect public perceptions of food safety. This highlights the need for government authorities to publicize provincial normative documents in various forms of media. For example, WeChat official accounts and blogs are the most popular online media in China and are thus able to aid in spreading normative documents to the general public. Perceived susceptibility is one of three dimensions of perceived risk [73] and we believe that better publicity for normative documents could help to mitigate residents’ risk perceptions of food safety [74]. In contrast, official communications in administration systems using bureaucratic language are not easily understandable to the general public in many cases [75]. To achieve barrier-free communication, our findings also suggest that provincial normative documents be written in plain language to enhance their readability.

### 5.2. Future Directions

Food safety is becoming one of the most important issues in people’s daily lives in developing countries. Thus, there are several research directions with great potential. Some of the provincial factors, including sample tests, have been suggested to influence public perceptions of food safety. We encourage researchers to test more provincial factors in future studies. It is also important to include provincial dummies (i.e., province, municipality, and autonomous region) and population size as explanatory variables. Additionally, it is possible that provincial economic states, government trust, number of food scandals, and the public’s media preference also have an effect on public perceptions of food safety. Meanwhile, we employed only one item to gauge residents’ overall perceptions of food safety, multiple item measures—for example, bacterial risk, excessive pesticide and nutrition loss—can also be included in further studies. In this study, we examined the provincial factors of residents’ overall perception of food safety national wide. Because the foods include local produced foods and nonlocal produced foods, it is worthwhile to examine how provincial regulations affect people’s food safety perception of foods with different producing areas.

Additionally, the demographic characteristics that influence residents’ perceptions of food safety should be considered from several levels. On one hand, political attitude, media preference, and social participation are also suggested to be included in follow-up studies. Demographic variables might moderate the relationships between the provincial factors and public perceptions of food safety. For example, since well-educated people might have better reading ability than less-educated people, we suppose the education background of residents moderates the effects of normative documents on perceptions of food safety. Last but not least, the influence of more regime tiers could be examined. In this paper, provincial factors were tested, but city-tier and county-tier analyses could allow more extensive observations since regulatory authorities at various tiers have different administrative privileges.

## Figures and Tables

**Table 1:** Local residents’ perception of food safety status quo in different provinces.

Province	Scores for Food Safety Perception	Province	Scores for Food Safety Perception
Anhui	5.383	Jilin	5.5
Beijing	5.176	Jiangsu	4.96
Fujian	5.421	Jiangxi	5.552
Gansu	6.222	Liaoning	5.162
Guangdong	5.364	Inner Mongolia	4.655
Guangxi	5.735	Shandong	5.923
Guizhou	6.547	Shanxi	5.673
Hainan	5.18	Shaanxi	4.94
Hebei	4.196	Shanghai	4.875
Henan	5.144	Sichuan	5.585
Heilongjiang	5.648	Yunnan	6.888
Hubei	5.598	Zhejiang	5.071
Hunan	5.568	Chongqing	5.421
Variance	0.314	SD.	0.549

**Table 2:** Descriptive statistics of demographic variables.

Variable	Definition	Frequency	Percentage (%)
Gender	Female = 0	1660	49.64
	Male = 1	1686	50.36
Age group	18–29 years = 1	610	18.40
	30–39 years = 2	507	15.16
	40–49 years = 3	711	21.17
	50–59 years = 4	655	19.54
	60 years and above = 5	863	25.73
Marriage	Unmarried, widowed, separated or divorced = 0	564	17.00
	Married or cohabiting = 1	2782	83.00
Child	No	1939	57.90
	Yes	1407	42.10
Education level	Primary school and below = 1	1303	38.89
	Secondary middle school = 2	1059	31.65
	Senior high school = 3	566	16.92
	College and above = 4	418	12.54
Household registration (Hukou)	Rural household registration = 1	2480	74.10
	Urban household registration = 2	866	25.90

**Table 3:** Descriptive statistics of regulatory variables.

Variables	Min	Max	Average	Standard Deviation
Normative documents (docs)	1	32	9.35	7.29
Sampling test (tests)	1152	32,594	8885.12	6749.98
Fiscal expenditure on food safety (per capita)	137	7309	1494.38	2670.92
Perception of food safety status quo	4.32	6.65	5.41	0.54

**Table 4:** Regression coefficients in hierarchical linear models.

Variables	Model 1	Model 2	Model 3	Model 4	Model 5	Model 6
Normative documents			−0.003			−0.016
			(0.007)			(0.009)
Sample inspection				0.183 *		0.222 *
				(0.088)		(0.085)
Fiscal expenditure (per capita)					0.175	0.126
					(0.158)	(0.146)
Gender		0.312 ***	0.312 ***	0.312 ***	0.31 ***	0.312 ***
		(0.078)	(0.077)	(0.077)	(0.077)	(0.076)
Age		0.261 ***	0.21 ***	0.26 ***	0.261 ***	0.26 ***
		(0.044)	(0.044)	(0.044)	(0.044)	(0.044)
Marital status		−0.26	−0.264	−0.24	−0.259	−0.259
		(0.150)	(0.151)	(0.141)	(0.149)	(0.145)
Number of children		−0.027	−0.026	−0.028	−0.028	−0.027
		(0.036)	(0.037)	(0.037)	(0.036)	(0.037)
Education level		−0.452 ***	−0.451 ***	−0.452 ***	0.452 ***	−0.45 ***
		(0.04)	(0.040)	(0.041)	(0.041)	(0.04)
Hukou		−0.673 ***	−0.675 ***	−0.651 ***	−0.68 ***	−0.656 ***
		(0.146)	(0.144)	(0.146)	(0.146)	(0.146)
Intercept of fixed effects	5.45 ***	6.751 ***	6.712 ***	5.053 ***	6.763 ***	4.898 ***
	(0.101)	(0.295)	(0.284)	(0.794)	(0.29)	(0.776)
Level-II variance component	0.206	1.182	1.204	1.144	1.205	1.246 *
Level-I variance component	6.111	5.393	5.393	5.389	5.392	5.389
Level 1 Units	3358	3358	3358	3358	3358	3358
Level 2 Units	26	26	26	26	26	26

Note: the significance level: * *p* < 0.05, ** *p* < 0.01, *** *p* < 0.001.
